# An Optimized Stimulation Control System for Upper Limb Exoskeleton Robot-Assisted Rehabilitation Using a Fuzzy Logic-Based Pain Detection Approach

**DOI:** 10.3390/s24041047

**Published:** 2024-02-06

**Authors:** Ismail Ben Abdallah, Yassine Bouteraa

**Affiliations:** 1 Control and Energy Management Laboratory (CEM Lab.), Ecole Nationale d’Ingénieurs de Sfax (ENIS), University of Sfax, Sfax 3038, Tunisia; ismail.benabdallah@enis.tn; 2 Department of Computer Engineering, College of Computer Engineering and Sciences, Prince Sattam Bin Abdulaziz University, Al-Kharj 11942, Saudi Arabia

**Keywords:** rehabilitation robotics, fuzzy classifier, pain detection, electrical stimulation, EMG signal-based control

## Abstract

The utilization of robotic systems in upper limb rehabilitation has shown promising results in aiding individuals with motor impairments. This research introduces an innovative approach to enhance the efficiency and adaptability of upper limb exoskeleton robot-assisted rehabilitation through the development of an optimized stimulation control system (OSCS). The proposed OSCS integrates a fuzzy logic-based pain detection approach designed to accurately assess and respond to the patient’s pain threshold during rehabilitation sessions. By employing fuzzy logic algorithms, the system dynamically adjusts the stimulation levels and control parameters of the exoskeleton, ensuring personalized and optimized rehabilitation protocols. This research conducts comprehensive evaluations, including simulation studies and clinical trials, to validate the OSCS’s efficacy in improving rehabilitation outcomes while prioritizing patient comfort and safety. The findings demonstrate the potential of the OSCS to revolutionize upper limb exoskeleton-assisted rehabilitation by offering a customizable and adaptive framework tailored to individual patient needs, thereby advancing the field of robotic-assisted rehabilitation.

## 1. Introduction

Upper limb exoskeleton robot-assisted rehabilitation poses several challenges in designing and implementing effective interventions for patients. One of the major challenges is the lack of perception in the rehabilitation training process, which limits the effect and safety of rehabilitation training [1]. To improve human–robot interactions in upper limb exoskeleton robot-assisted rehabilitation, a proposed motion intensity perception model based on deep neural networks can be used to address this challenge [1,2]. Another challenge is designing rehabilitation training tasks according to the situation of patients [3]. Upper limb exoskeleton robot-assisted rehabilitation requires optimizing human–robot interactions for better performance [4]. The use of predictive dynamics (PD) is essential in determining unique paths for each exoskeleton joint while accounting for the range of motion of the human arm [5]. In order to facilitate this, the design of the exoskeleton ought to be in line with the kinematics of the human arm, integrating autonomous revolute joints that permit complete arm motion [6]. In order to support the rehabilitation process, the system must also keep motion logs for data analysis. In order to provide thorough monitoring and analysis, it must additionally record and preserve specific joint movements [7]. Users could participate in daily activities with a full range of motion and enhance training sessions with an anthropomorphic design that promotes natural and intuitive engagement [5]. The paramount design considerations encompass safety, reliability, wearability, portability, and user friendliness, ensuring the movement of anatomical centers of rotation during locomotion, along with independent joint control. Upper limb exoskeleton robot-assisted rehabilitation is a field of study with multiple modalities, including passive, assistive, and active-assistive modes, and several popular robot-mediated devices exist in the field of study, including BONES, KINARM, L-Exos, and MAHI EXO-II [8,9,10,11]. However, evaluating outcomes based on abilities influencing the quality of life in upper limb exoskeleton robot-assisted rehabilitation is often not objective enough, making it difficult to compare the results of clinical controlled trials and implement effective interventions [12].

The optimization of stimulation control systems in upper limb rehabilitation robotic systems is crucial for effective and safe rehabilitation training. Fuzzy logic-based pain detection approaches have been proposed as one solution to optimize these systems. For example, the RAX-1 rehabilitation robotic system comprises two joints that facilitate shoulder joint movements [13]. The proposed method has also been investigated for rehabilitation purposes for both upper and lower limb exoskeletons, demonstrating its applicability in multiple settings [14]. Other studies have focused on the design and evaluation of surface electromyography-controlled lightweight upper arm exoskeleton rehabilitation robots, highlighting the potential benefits of utilizing fuzzy logic-based pain detection approaches in optimizing stimulation control systems in such robots [15]. To maximize the efficacy of upper limb rehabilitation robots, various strategies are being employed. These encompass customizing trajectories, optimizing movements, and implementing tracking control algorithms, particularly in the context of a novel upper limb rehabilitation robot [16]. Researchers have also introduced designs centered around exoskeletons for robot-assisted rehabilitation, aligning with the kinematics of the human arm [5,17]. Furthermore, a highly integrated upper extremity rehabilitation robot that hinges on a human–machine coupling system model, recognizing the pivotal role of the shoulder joint in rehabilitation, has been proposed [18]. Studies demonstrate the effectiveness of robotic rehabilitation systems in addressing upper extremity paralysis post-stroke [19]. Efforts to enhance rehabilitation training and safety involve exploring novel designs and control methods for human–exoskeleton robot interactions [1]. Consequently, employing fuzzy logic-based pain detection approaches holds substantial promise in optimizing stimulation control systems within upper limb rehabilitation robots, ultimately leading to improved rehabilitation outcomes and heightened patient safety.

The goal of integrating EMG-based myoelectric control systems into exoskeletons is to promote better human–robot interactions and increase adaptability in a variety of motion tasks [20]. Interestingly, these solutions show promise for improving performance in those without mobility problems [20]. Furthermore, wearable upper limb robotic exoskeletons with sophisticated myoelectric control systems demonstrate encouraging advancements in rehabilitation by assisting individuals with motor disorders with moving and regaining their motor functions [21]. The supported degrees of freedom, portability, and particular application situations are important considerations in the design of myoelectric controllers since they have a substantial impact on the controller’s efficacy [22]. While current myoelectric control systems have limitations, future research directions can overcome these challenges [23]. In terms of rehabilitation safety, an ABC-optimized PID controller demonstrated better performance in response characteristics compared to the Ziegler–Nichols method, highlighting the importance of optimized stimulation control systems for robustness and safe rehabilitation [24]. Furthermore, a controller optimized with ABC showed minimal overshoot in response and significantly low response times, which are desirable in rehabilitation. Such an optimized stimulation control system can ensure steady motion with no steady-state error during the rehabilitation process while reducing the risk of joint dislocation [24]. Thus, the use of an optimized stimulation control system is essential for upper limb exoskeleton robot-assisted rehabilitation to improve efficacy and patient safety.

**Figure 1:**
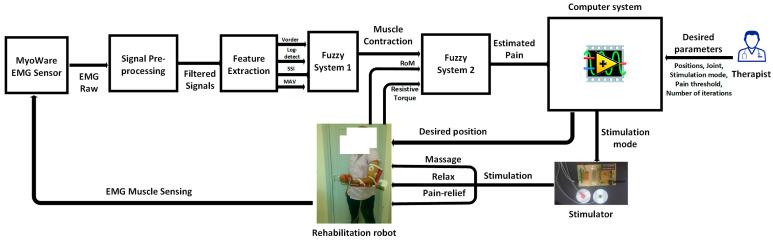
System overview.

This study introduces a novel method aimed at improving the effectiveness and flexibility of upper limb exoskeleton robot-assisted rehabilitation. The innovation lies in the development of an optimized stimulation control system (OSCS). This OSCS incorporates a fuzzy logic-based pain detection approach specifically engineered to accurately gauge and respond to a patient’s pain tolerance during rehabilitation sessions. Through the utilization of fuzzy logic algorithms, as shown in Figure 1, the system dynamically adjusts the exoskeleton’s stimulation levels and control parameters. This ensures personalized and fine-tuned rehabilitation protocols. This research conducts extensive assessments, encompassing simulation studies and clinical trials, to validate the OSCS’s efficiency in enhancing rehabilitation outcomes while giving utmost priority to patient comfort and safety. The results highlight the OSCS’s potential to transform upper limb exoskeleton-assisted rehabilitation by providing a tailored, adaptable framework that caters to individual patient requirements. This advancement signifies a significant step forward in the realm of robotic-assisted rehabilitation.

## 2. Materials and Methods

### 2.1. Mechatronic Design

The design prerequisites for upper limb exoskeleton robots necessitate meticulous considerations in both mechanical and electrical domains to ensure optimal functionality, safety, and user adaptability. On the mechanical front, the exoskeleton’s structural design prioritizes lightweight yet robust materials, such as carbon fiber composites or high-strength alloys, striking a balance between durability and minimizing weight to alleviate user fatigue. A detailed biomechanical analysis guides joint configurations and linkages, replicating natural human movement while ensuring sufficient strength to support and augment the user’s limb motion.

Precise actuation mechanisms and joint articulation are fundamental mechanical components. Robust yet compact actuators, such as electric motors or pneumatic systems, are strategically placed along the exoskeleton to facilitate smooth and responsive movements. These actuators must deliver adequate torque and force to support a range of motions without compromising the exoskeleton’s overall weight and agility. The joint mechanisms are designed for compliance and adaptability, allowing natural movement patterns while offering support or resistance as needed. This mimics and enhances the user’s capabilities.

From a design perspective, our objective is to create a high-precision rehabilitation system that accommodates two primary constraints: portability and weightlessness. Initially, extensive research was undertaken to identify the most suitable actuators for the system. The primary criterion considered was the motor torque necessary to lift the entire upper limb. While DC motors coupled with gearboxes offer adequate torque, they lack positional accuracy, necessitating the inclusion of rotary encoders. However, this solution was disregarded due to space constraints. Alternatively, stepper motors provide moderate movement precision but with relatively low torque. Although high-torque variants are available, they come at a significant cost. To address space-related issues and enable easy integration and fixing, we opted for precise feedback servomotors. Despite their advantages, these servomotors face limitations concerning torque. Our solution involves employing geared servomotors, mitigating the torque issue while leveraging the benefits of seamless integration and precise fixing without backlash.

**Figure 2:**
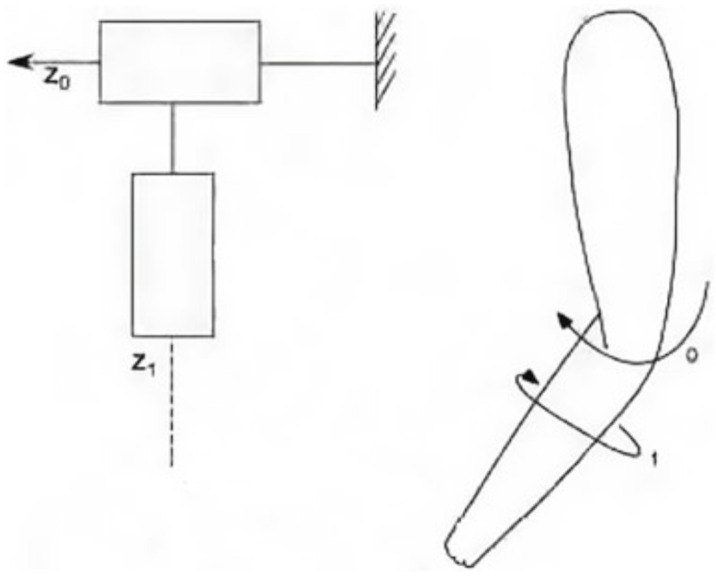
Kinematic model of the elbow-forearm movement.

The elbow-forearm exoskeleton is designed with the goal of imitating the natural arm shape, with an emphasis on the operator’s workspace and the preservation of normal arm motion. As seen in Figure 2, the exoskeleton’s basic kinematic structure consists of two revolute joints: one for elbow movement (Axis 0) and another for forearm rotation (Axis 1). One of the most important aspects of the exoskeleton is its torque-generating ability, which complements and enhances human capabilities. Table 1 provides a full overview of the workspace and torque capabilities unique to the elbow-forearm rehabilitation system.

**Figure 3:**
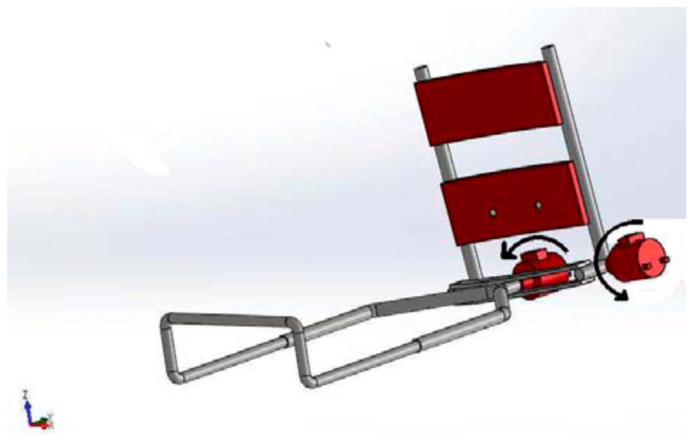
Three-dimensional design of elbow-forearm rehabilitation device.

As shown in Figure 3, the rehabilitation robot is made up of multiple essential mechanical parts. To ensure the general functionality of the robot, the upper limb support fits tightly onto the human arm, acting as an exoskeleton part. It is important that it be attached securely, either onto a solid support or directly onto the triceps using metal plates on one side and scratches on the other. The stainless-steel plates are shaped like an arc to fit the contours of the triceps. The wrist handle adds flexibility to the system by slidably connecting to the spindle and adjusting to the size of the patient’s arm. It also gives the patient’s hand a grip and makes pronation-supination movements easier. The second degree of freedom is the crucial pronation-supination joint movement, which is made possible by the connecting spindle, which is attached directly to the motor shaft. It also serves as a connecting element between the wrist handle and the intermediate metal plate. The intermediate metal plate allows rotational motions (flexion/extension) between the two portions by binding the upper limb support and forearm support. We offer multiple modes of use for convenience:

1. Portable mode involves a backpack carrying the electronic control board and connects to the power adapter, allowing the option of battery-powered operation.
2. Sitting position mode allows the user to sit on a chair while the rehabilitation exoskeleton is secured on a flexible-length supporting holder. A compact box placed on the supporting base houses the PCB, which is connected to an electrical outlet for power supply.

### 2.2. Stimulator Design

Designing a functional electrical stimulation (FES) system demands meticulous attention to various critical design requirements. Primarily, the FES system must exhibit precision in stimulating targeted muscles to elicit controlled and effective muscle contractions. The system’s capability to generate electrical impulses at precise timings, frequencies, and amplitudes is crucial, ensuring the synchronized activation of muscles for specific tasks or rehabilitation purposes. Moreover, safety considerations are paramount; the FES system should comply with stringent safety standards to prevent overstimulation or discomfort for the user. To enhance usability, the system should be ergonomic, offering intuitive interfaces for easy operation and adjustment of stimulation parameters. Versatility is also essential, enabling the FES system to adapt to different muscle groups or rehabilitation needs. Additionally, the system should be compact, portable, and energy efficient for practical use in various settings, such as clinical environments or home-based rehabilitation programs. Robustness and reliability are imperative, ensuring consistent performance and durability over extended periods. Furthermore, considerations for wireless connectivity, advanced control algorithms, and compatibility with other rehabilitation devices contribute to the comprehensive design requirements of a modern and effective FES system. Overall, a successful FES design harmonizes precision, safety, usability, adaptability, portability, durability, and technological innovation to offer targeted muscle stimulation for rehabilitation or functional enhancement purposes.

**Figure 4:**
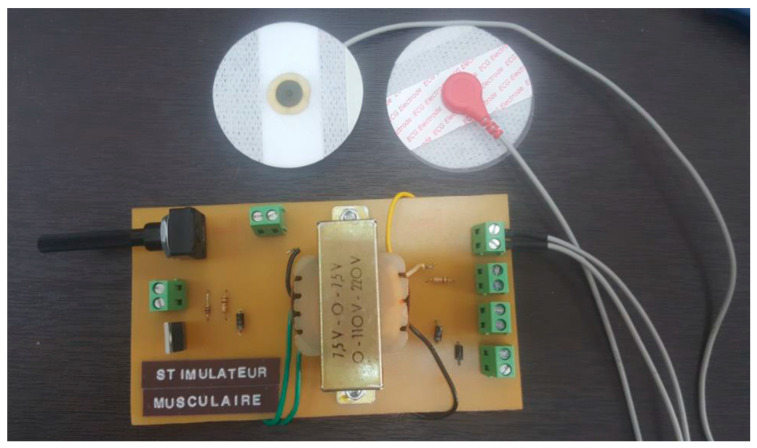
PCB of the lab-made stimulator.

The specialized stimulator developed in our laboratory (seen in Figure 4) is purpose-built to seamlessly integrate with an exoskeleton robot, featuring a sophisticated printed circuit board (PCB). Designed with intricate attention to detail, this PCB stimulator possesses a compact form perfectly suited for incorporation within the exoskeleton’s structure. Precision engineered, it houses premium electronic components strategically placed for precise control over stimulation parameters like voltage, frequency, and pulse width. Indeed, the output voltage can be adjusted by using the implemented potentiometer. On the other hand, frequency and pulse width can be adjusted via signals from a microcontroller. The main specifications of the designed stimulator are illustrated in Table 2. Its user-friendly interface empowers researchers to efficiently configure stimulation settings. The stimulator’s innovative design includes communication interfaces that foster seamless interaction between the exoskeleton robot and the stimulator, optimizing synchronization and control. Its adaptable nature and tailored integration offer a valuable tool for conducting targeted upper limb rehabilitation experiments, enhancing the study and refinement of robot-assisted rehabilitation protocols.

### 2.3. Control Architecture Design

#### 2.3.1. System Overview

Figure 1 showcases an integrated architecture designed to control a rehabilitation robot in tandem with a customized stimulator. At the heart of this framework is the therapist overseeing the entire rehabilitation procedure. The control system acts as a central hub, coordinating the actions of the stimulator, which interfaces with the rehabilitation robot.

This architecture utilizes EMG (electromyography) signals obtained through the Myoware sensor, capturing muscle activity during rehabilitation sessions. These signals undergo preprocessing via a specialized EMG filtering block, refining and preparing them for further analysis. Subsequently, feature extraction algorithms are employed to distill relevant information from the filtered EMG signals. These extracted features serve as inputs for a fuzzy classifier, a critical component used to accurately estimate the patient’s pain level.

The computer system assumes a vital role, leveraging the outcomes from the fuzzy classifier to autonomously regulate both the stimulator and the exoskeleton robot. This closed-loop system dynamically adjusts the rehabilitation process based on the patient’s estimated pain levels, enabling personalized and responsive therapy. Figure 1 encapsulates a complex yet coherent framework integrating EMG signal acquisition, preprocessing, feature extraction, pain estimation, and autonomous control of the rehabilitation robot and stimulator. Ultimately, this integration enhances the effectiveness and adaptability of the rehabilitation process.

#### 2.3.2. EMG Acquisition and Preprocessing

- Acquisition

In assessing pain levels during human–exoskeleton interactions, EMG recordings prove invaluable. This research focused on evaluating muscle contraction in two major arm muscles, the extensor digitorum longus (EDL) and the flexor digitorum longus (FDL). To acquire signals, AgCl electrodes were adhered to the surface of the EMG following the standards for noninvasive muscle evaluation as per the SENIAM guidelines [25]. To optimize signal capture, the participant’s skin was cleansed with an alcohol swab and shaved as recommended. Electrode placement adhered to the SENIAM guidelines, utilizing pre-gelled electrodes with a 20 mm inter-electrode interval for each bipolar derivation, with the reference electrode placed at the wrist.

With the use of surface electrodes, the Myoware EMG sensor made it easier to acquire signals by offering two outputs: raw and rectified EMG. With the exception of threshold detection, the rectified output—which uses an integrated analog conditioning block—provides time domain average EMG signal values but is unsuitable for additional digital processing. On the other hand, digital processing can benefit from the raw EMG output, making it possible to extract relevant features. The raw EMG was used as the acquisition system’s input in this investigation, along with the real-time board sbRIO-9637 from National Instruments.

The signal was amplified with a strength of 1000, 10,000 samples per second, and a sampling frequency of 100 kHz [26]. The band-pass filter used was a Butterworth type, with a frequency range of 10–2000 Hz [27]. Two channels of EMG signals were produced by placing electrodes on both sides of the relevant muscles and placing a reference electrode at the olecranon joint. In addition, a digital filter design module in LabVIEW was used to create a Butterworth-type notch filter at 50 Hz with the goal of removing power line noise and improving signal quality.

- EMG processing for pain estimation

Algorithms for pattern matching and signal processing have been developed to accurately interpret the information contained in the EMG signal. These algorithms include pattern classification and characteristic extraction. As opposed to FD (frequency domain) and TS (time-scale) features, which arise from the raw EMG signal, TD (time domain) features are the easiest to compute and apply. Additional transformations are required due to the computational difficulty of TS and FD features, which calls for specialized processors. In this study, the fuzzy logic controller’s first block—which estimates muscular contraction—primarily uses TD features as inputs. In order to extract muscle contraction from the EMG signal and analyze patient pain during rehabilitation sessions for safety precautions, this information is essential. Within the analysis timespan of N samples, four features are extracted based on similar previous studies investigating the selection of the EMG features for movement classification during muscle contraction [28,29,30].

The root mean square (*RMS*), a metric linked to both constant force and non-fatiguing muscular contractions, is described using the amplitude-modulated Gaussian random process:

(1) $$RMS=\sqrt{\frac{1}{N}}\sum_{i=1}^{N}X_{i}^{2}$$

where *X* is the signal.

One useful aspect that expresses the energy of the EMG signal is the simple square integral (*SSI*):

(2) $$SSI=\sum_{i=1}^{N}\left|X_{i}\right|$$

An estimation of the force applied to the muscles is given by the EMG_*vorder*:

(3) $$vorder=\sqrt{E\left|X_{k}\right|}$$

where *E* is the expectation operator applied on simples in one analysis window.

An estimation of the force applied to the muscles is given by the *Log detector*:

(4) $$Logdetect=e^{\frac{1}{N}}\sum_{k=1}^{N}log(\left|X^{i}\right|)$$

#### 2.3.3. Fuzzy Logic-Based Pain Estimation

Pain sensing during robotic rehabilitation holds paramount importance in ensuring safe and effective therapy sessions. Integrating pain-sensing mechanisms within robotic rehabilitation systems allows for real-time monitoring of a patient’s discomfort or pain levels during exercises or assisted movements. This capability enables the system to adapt and personalize the rehabilitation process based on the patient’s comfort thresholds, preventing potential injuries or exacerbation of discomfort. Pain sensing enhances the safety and efficacy of rehabilitation by allowing immediate adjustments in intensity, range of motion, or exercise type if the patient experiences discomfort, ensuring that the therapy remains within safe and tolerable limits. By incorporating pain-sensing technologies, robotic rehabilitation systems can provide tailored and responsive assistance, promoting patient confidence, comfort, and overall rehabilitation outcomes. Moreover, pain sensing helps therapists and healthcare professionals with objectively assessing a patient’s progress, facilitating the development of individualized rehabilitation plans that consider pain management as an integral part of the overall treatment strategy.

**Figure 5:**
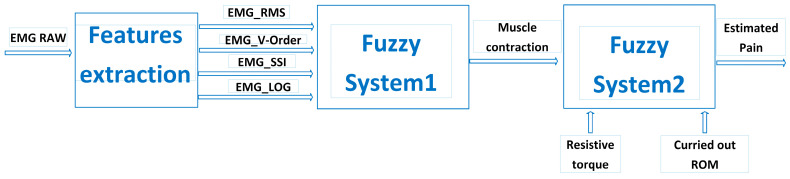
The fuzzy system architecture.

Within the rehabilitation realm, the precise pain threshold often remains an approximation rather than an exact value. Consequently, a concept of partial reality becomes crucial, where decisions are derived from a set of fuzzy constraints. The occurrence of a current peak, typically stemming from a subject’s resistance force, does not definitively confirm severe pain, as it might result from various factors. However, when this peak coincides with muscular contraction, it strongly indicates the subject’s pain level. Surprisingly, pain itself may not always lead to movement cessation or alterations; at certain rehabilitation stages, intense pain may be a natural symptom. A fuzzy decision support system needs to include a third input function for final decision making in order to handle this complexity. The patient’s resistance and the previously attained range of motion (RoM) are integrated by the fuzzy system in the suggested architecture. Even in the face of terrible pain, the robot is able to proceed with rehabilitation thanks to this fuzzy logic framework as long as the recovery objective has been met in the past. The system is made up of two blocks: the first uses data from the EMG sensor (EMG_RMS, EMG_SSI, EMG_vorder, and EMG_logdetect) to estimate muscle contraction, and the second evaluates pain intensity based on patient resistance, muscle contraction, and the most recent RoM. The fuzzy architecture that is provided is seen in Figure 5.

An integrated current sensor on the motor driver board serves the purpose of approximating the passive torque associated with the respective joint. Essentially, this passive torque corresponds to the torque produced by the motor, and its estimation relies on the analysis of the consumed current. The correlation between motor current and the resultant torque is determined through the following conventional equation:

(5) $$T\left(Nm\right)=K_{t}*I\left(A\right)$$

wherein K_t_ represents the constant torque supplied by the motor’s manufacturer.

To monitor pain levels and control the rehabilitation system, a fuzzy system was developed using the LabVIEW PID and Fuzzy Logic Toolkit. The input and output membership functions are represented as Gaussian shapes. Furthermore, the center of area method was employed for de-fuzzification purposes. Given that EMG constitutes a bio-signal contingent upon individual subjects and is notably influenced by measurement conditions and electrode placement, normalization of EMG data is crucial. This involves mapping the time domain parameters to ensure uniformity and comparability across different subjects’ EMG readings.

The following formula provides the mapping equation:

(6) $$Y_{N}=\frac{\left(Y_{Nmax}-Y_{Nmin})(Y-Y_{min}\right)}{\left(Y_{max}-Y_{min}\right)}+Y_{min}$$

where [$Y_{min}$,$Y_{max}$] is the range of the input parameters *Y* before mapping and [$Y_{Nmin},Y_{Nmax}]$ is the output range of the normalized parameter $Y_{N}$. The normalized time domain parameters range from 0 to 10.

The center of area method was applied for defuzzification in this research. Consequently, the geometric center of this region was determined by the fuzzy logic controller using the following equation:

(7) $$CoA=\frac{\int_{X_{min}}^{X_{max}}f\left(x\right)*x\;dx}{\int_{X_{min}}^{X_{max}}f\left(x\right)\;dx}$$

When *x* represents the linguistic variable’s value, *x_min_* and *x_max_* denote the variable’s range, and *CoA* is the area’s center.

**Figure 6:**
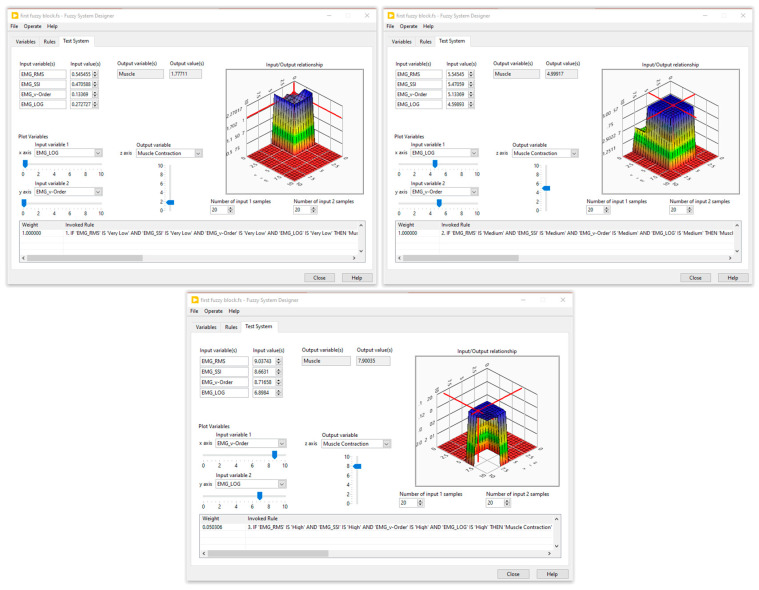
Simulation results of the first fuzzy block.

**Figure 7:**
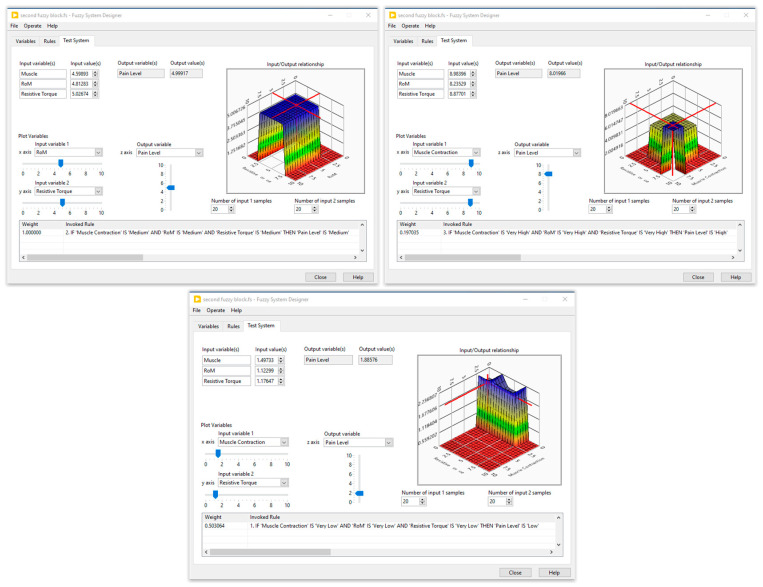
Simulation results of the second fuzzy block.

Five membership functions were created and given the following names for each input of the first fuzzy system: very low, low, medium, high, and very high. Furthermore, three membership functions—low, medium, and high—were set aside for the generation of muscle contractions. The second fuzzy block contained three inputs, the muscle contraction (three membership functions), the resistive torque (five membership functions), and the carried out RoM (four membership functions), while the output was designed with four membership functions. The fuzzy rules were defined by an expert in the field of the rehabilitation. Several tests on healthy subjects were performed to optimize these rules. The simulation results of the designed fuzzy systems are illustrated in Figure 6 and Figure 7. 

## 3. Results and Discussion

The upper limb device suggested here is made up of three segments: the arm, forearm, and wrist. It is intended to accommodate all normal movements that are connected to the elbow and forearm joints. The two main movement types of the upper limb are flexion/extension and pronation/supination. Flexion and extension change joint angles by bringing limb segments together or lining them up, whereas pronation and supination rotate the hand; supination rotates the hand externally (palm upward), and pronation rotates it downward (internally). The upper limb rehabilitation robot’s design must adhere to joint standards.

**Figure 8:**
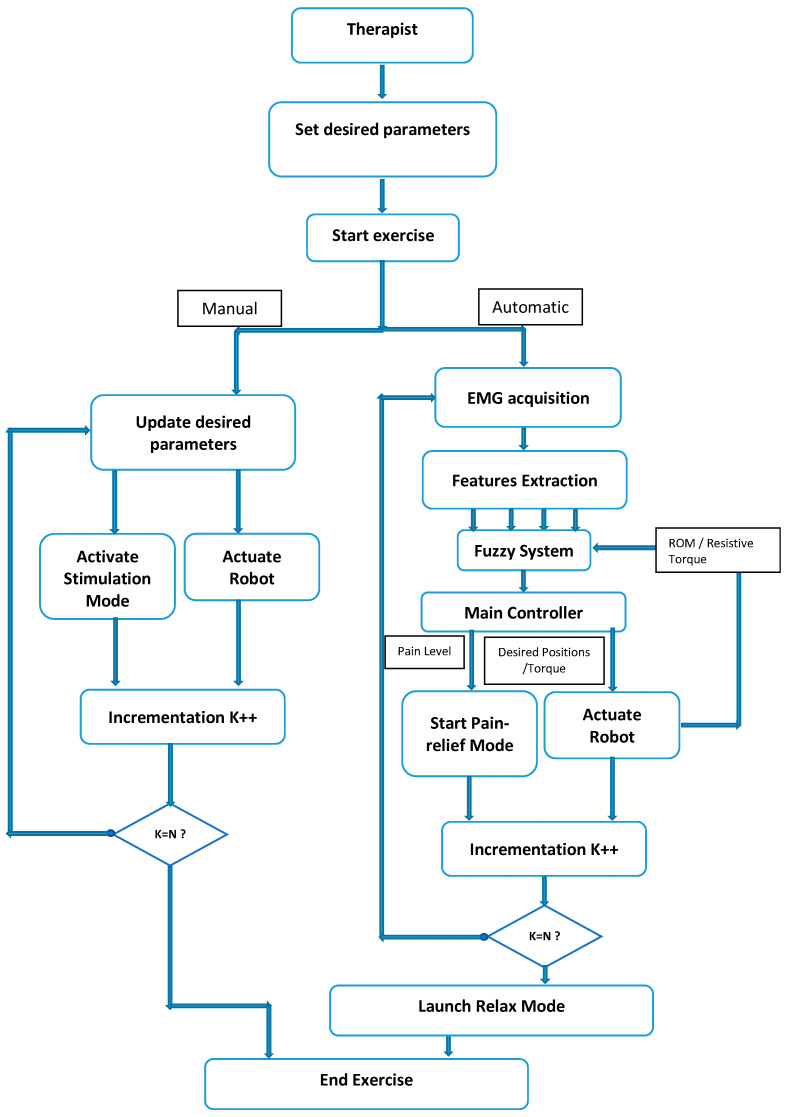
Flowchart.

During the upper limb rehabilitation session employing a rehabilitation robot and electrical stimulator, the physiotherapist initiates the process by inputting specific parameters into a LabVIEW-based human–machine interface (Figure 8). This action triggers the start of the rehabilitation exercise. Initially, the therapist selects the stimulation mode, which can either be manual or automatic. In manual mode, the computer system updates the parameters, executes the chosen stimulation mode, and operates the rehabilitation robot for the designated number of iterations. The therapist retains control to manually enable any stimulation mode based on the patient’s condition. Conversely, the automatic mode relies on acquired EMG signals to gauge the patient’s muscle state in real time. A fuzzy controller estimates muscle pain by analyzing muscle features, range of motion, and joint torque. The main controller then adjusts the rehabilitation robot and stimulator based on this pain estimation, enabling the exercise without direct therapist intervention. Once the predetermined cycles conclude, the stimulator transitions automatically to a relaxation mode. It is highly recommended for the physiotherapist to use the manual mode before moving to the automatic mode. Indeed, the physiotherapist can use the manual test to understand the main features of the designed rehabilitation system. By supervising the feedback of the patient in each stimulation mode, the physiotherapist can select the suitable pain level threshold, which will be used in the automatic mode.

**Figure 9:**
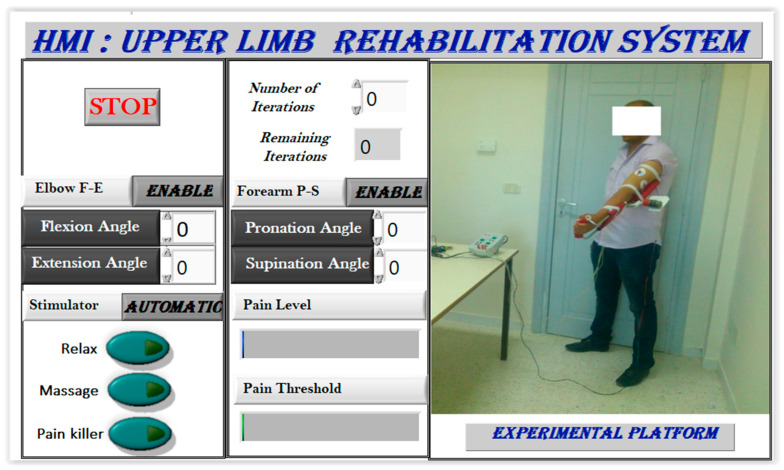
Blank user interface.

**Figure 10:**
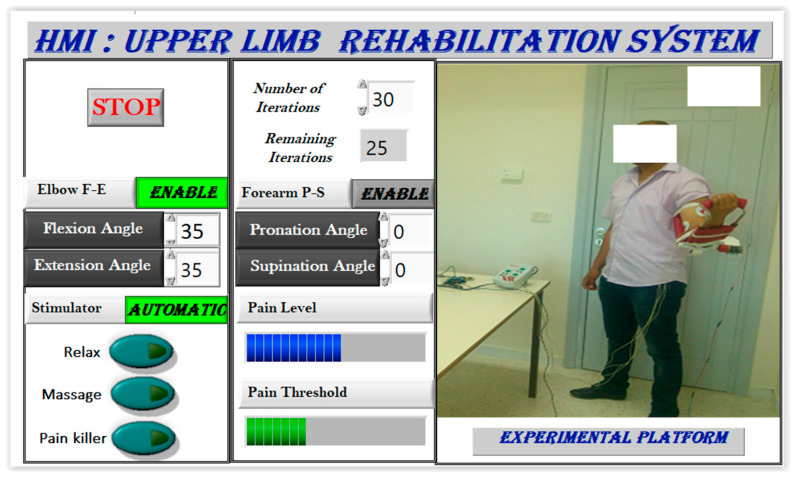
Executed user interface.

Figure 9 showcases the human–machine interface (HMI), which functions as a configuration and supervision tool with various control and supervision fields. The software (LabVIEW 2020), illustrated in Figure 10, offers two stimulation activation modes: manual/predefined and automatic. In the manual mode, the physiotherapist presets stimulation before motor rehabilitation, while the automatic mode selects stimulation based on measured EMG signals. During exercises, time domain EMG signals are acquired, processed to extract useful features, and utilized for pain detection, activating pain relief or massage modes. The relaxation mode follows each rehabilitation protocol, offering relief and reducing post-exercise pain.

The user interface allows joint position control while supervising estimated pain levels through EMG signals, enriching rehabilitation protocol options. The automatic stimulation mode enhances system autonomy by selecting suitable stimulation based on muscular responses. These stimulation modes aid in revitalizing muscle responses and fostering relevant progress during rehabilitation sessions. Integrating electrical stimulation into motor rehabilitation aims to enable enduring pain tolerance, ensuring completion of pre-programmed exercises. Electrical stimulation can alleviate various pain types (neuropathic, nociceptive acute, or chronic) by triggering analgesic and endorphin effects on sensory nerve fibers, akin to muscle contractions initiated by the brain. Electrical stimulation directly excites motor nerves with optimal impulses, ensuring efficacy and safety in muscle excitation.

Two weeks after the plaster was removed, a patient with a fractured forearm underwent extensive therapy with the goal of restoring flexion/extension movements. As passive mobilization is more tolerable than manual approaches, the aim was to guarantee that rehabilitation was painless, gradual, and constant. In order to activate the pain alleviation mode based on obtained EMG data, each exercise was performed five times, for a total of thirty iterations. Upon completion of the session, the relax mode was automatically activated. The individual had a roughly 60-degree elbow range of motion on 30 July 2023, when they were first evaluated. The robotic system significantly increased the range of motion (RoM) to 85 degrees during the initial rehabilitation session. This improvement was observed on average after 50 repetitions of flexion/extension movement, which is equivalent to one degree. Over two weeks, the developed system significantly enhanced the elbow’s RoM, reaching 110 degrees. However, upon analyzing RoM against iterations, it was evident that the improvement rate had slowed down, approaching levels comparable to a healthy subject’s performance. 

The subject experienced an approximate 83% increase in joint RoM compared to the initial state (see Table 3), validating the robot’s efficacy as a valuable tool for improving the range of motion, exhibiting competitive performance compared to recent similar works [26,27,31,32]. Moreover, the evaluation of estimated joint torque showcased a 30% reduction in passive torque, an aspect scarcely reported in recent research on human joint progress post-robotic rehabilitation training [33,34,35,36].

Electromyography (EMG) signals have shown potential for detecting muscle state during robotic rehabilitation training in physical therapy. Indeed, rehabilitation robots equipped with reactive electromyography capture EMG signals from a patient’s muscles, allowing them to assist in the rehabilitation process [37]. Studies have investigated the use of EMG signals in robot-based stroke neurorehabilitation to improve functional outcomes for patients [38]. To address the shortcomings of traditional medical rehabilitation evaluation methods, a musculoskeletal evaluation system based on EMG has been proposed, which provides a more accurate assessment of muscle function and pain relief [39]. Electromyography (EMG) is also being developed for stroke rehabilitation to enhance motion control in clinical settings [40]. Finally, studies on adaptive robot-assisted upper limb training interactions have not always considered the implications of muscle pain sufficiently, highlighting the importance of EMG signals in monitoring muscle activity during rehabilitation sessions [41].

While many recent studies have focused on muscle state estimation during rehabilitation training [42,43,44], integrating muscle pain level into the primary controller remains a challenge. Moreover, only a few studies have reported on muscle pain estimation based on the acquired EMG signals [45,46,47,48,49]. The pain level can be estimated using facial expression [45,46] or during walking [47,48]. Typically, when muscle contraction is sensed, rehabilitation protocols manually halt or adjust exercises for patient protection. This paper demonstrates the design of a fuzzy classifier and selection of appropriate feature extraction methods to highlight the classifier’s high performance. 

## 4. Conclusions

The designed rehabilitation system incorporates two distinct rehabilitation techniques, motor rehabilitation and electrical stimulation, aiming to leverage the advantages of both methodologies. Equipped with a customized stimulator capable of delivering three stimulation modes—pain relief, massage, and relaxation—the system’s software offers two activation modes: manual preset and an innovative EMG-driven mode. In the latter, the stimulator responds to the EMG signal, activating one of its three modes accordingly. Rigorous experimental tests conducted on the robot showcased flawless system functionality. An initial case study highlighted the system’s effectiveness in terms of rehabilitation and monitoring. While the encouraging results of the newly designed rehabilitation system showcase its potential, it is essential to acknowledge the limitations associated with the current study, which involved only a single subject. The efficacy and generalizability of the developed rehabilitation system may be influenced by individual variations and specific characteristics of the sole participant. To validate and strengthen the reliability of the system, future research endeavors should encompass a more extensive and diverse range of case studies, specifically those involving individuals who have experienced strokes. This broader inclusion of subjects would provide a more comprehensive evaluation of the rehabilitation system’s performance across varying conditions and user profiles. Additionally, such a multi-case approach is crucial for establishing the robustness and applicability of the system in diverse clinical scenarios, ultimately contributing to its wider acceptance and effectiveness in stroke rehabilitation.

## Figures and Tables

**Table 1:** Specifications of the designed robot.

Joint	Motor Type	Gear Type	Torque (Nm)	Backlash	Workspace (Degree)
Elbow	Geared Servomotor	Planetary	65	<0.1°	Flexion: 130/Extension: 0
Forearm	Geared Servomotor	Planetary	8	<0.1°	Supination: 90/Pronation: 90

**Table 2:** Main specifications of the designed stimulator.

Features	Specifications
Output Mode	3 modes (pain relief, massage, and relax)
Pulse Amplitude	Adjustable: up to 80 v
Pulse Rate	Adjustable: 1~100 Hz
Pulse Width	Adjustable: 20~300 μs
Channels	4 channels

**Table 3:** Obtained results.

	Joint	Stimulation	EMG Classification Algorithm	Recovery Percentage (RoM)	Recovery Percentage (Passive Torque)
Our research	Elbow	3 modes	Fuzzy	83%	30%
Ref [26]	Forearm	NA	SVM	35%	28%
Ref [27]	Wrist	NA	Fuzzy	22%	19%

## Data Availability

Data are contained within the article.
